# Genetic mapping revealed that the *Pun2* gene in *Capsicum chacoense* encodes a putative aminotransferase

**DOI:** 10.3389/fpls.2022.1039393

**Published:** 2022-11-01

**Authors:** Shieun Yi, Do-Gyeong Lee, Seungki Back, Ju-Pyo Hong, Siyoung Jang, Koeun Han, Byoung-Cheorl Kang

**Affiliations:** Department of Agriculture, Forestry, and Bioresources, Research Institute of Agriculture and Life Science, Plant Genomics and Breeding Institute, College of Agriculture and Life Science, Seoul National University, Seoul, South Korea

**Keywords:** Capsaicinoid biosynthesis, *Pun2*, *pAMT*, *Capsicum chacoense*, QTL mapping

## Abstract

Several genes regulating capsaicinoid biosynthesis including *Pun1* (also known as *CS*), *Pun3*, *pAMT*, and *CaKR1* have been studied. However, the gene encoded by *Pun2* in the non-pungent *Capsicum chacoense* is unknown. This study aimed to identify the *Pun2* gene by genetic mapping using interspecific (*C. chacoense* × *Capsicum annuum*) and intraspecific (*C. chacoense* × *C. chacoense*) populations. QTL mapping using the interspecific F_2_ population revealed two major QTLs on chromosomes 3 and 9. Two bin markers within the QTL regions on two chromosomes were highly correlated with the capsaicinoid content in the interspecific population. The major QTL, *Pun2_PJ_Gibbs_3.11* on chromosome 3, contained the *pAMT* gene, indicating that the non-pungency of *C. chacoense* may be attributed to a mutation in the *pAMT* gene. Sequence analysis revealed a 7 bp nucleotide insertion in the 8^th^ exon of *pAMT* of the non-pungent *C. chacoense*. This mutation resulted in the generation of an early stop codon, resulting in a truncated mutant lacking the PLP binding site, which is critical for pAMT enzymatic activity. This insertion co-segregated with the pungency phenotype in the intraspecific F_2_ population. We named this novel *pAMT* allele *pamt^11^*. Taken together, these data indicate that the non-pungency of *C. chacoense* is due to the non-functional *pAMT* allele, and *Pun2* encodes the *pAMT* gene.

## Introduction

Pepper (*Capsicum* spp.) is the only natural resource producing the pungency principle capsaicinoid. Capsaicinoids are secondary metabolites in pepper that function as protection against insects and microorganisms (Tewksbury and Nabhan, 2001; Levey et al., 2006). Capsaicinoids have been used as food additives, medicine, and personal defense tools due to their pungency and their pharmacological and chemical effects (Meghvansi et al., 2010; Khan et al., 2014; Srinivasan, 2016). Some pepper cultivars produce capsinoids, which have similar chemical structure and properties to capsaicinoids, but these are almost non-pungent (Tanaka et al., 2009). Capsinoids as well as capsaicinoids have been proven to exhibit anti-cancer, anti-obesity, anti-diabetic, and antioxidant activities (Uarrota et al., 2021).

Capsaicinoids are conjugated compounds of precursors derived from the phenylpropanoid pathway and the branched-chain fatty acid pathway. In the phenylpropanoid pathway, phenylamine is converted to precursors by phenylalanine ammonia-lyase (PAL), cinnamate 4-hydroxylase (C4H), 4-coumaroyl-CoA ligase (4CL), *p*-coumaroyl shikimate/quinate 3-hydroxylase (C3H), caffeoyl-CoA 3-*O*-methyltransferase (COMT), and putative aminotransferase (pAMT), resulting in vanillylamine (Bennett and Kirby, 1968; Leete and Louden, 1968; Sukrasno and Yeoman, 1993; Curry et al., 1999). In the branched-chain fatty acid pathway, the final products are produced from either valine or leucine by a series of enzymes including acetolactate synthase (ALS), branched-chain amino acid aminotransferase (BCAT), ketoacyl-ACT synthase (KAS), acyl carrier protein (ACL), ketoacyl-ACP reductase (KR1), and acyl-ACP thioesterase (FAT). The products of the two pathways, vanillylamine and the branched-chain fatty acids, are condensed by capsaicin synthase (CS), an acyltransferase 3 (AT3) also known as Pun1, resulting in different capsaicinoids depending on the type of branched-chain fatty acids (Suzuki et al.,, 1981; Harwood, 1996; Markai et al., 2002; Koeda et al., 2019).

Although capsaicinoid biosynthesis has long been studied, the structural genes involved in the pathway have not been fully elucidated (Aza-González et al., 2011). The *Pun1* gene was considered the only gene that affects the loss of pungency in pepper until two other loci, *Pun2* and *Pun3*, were identified (Stellari et al., 2010; Han et al., 2019). *Pun1* encodes a putative acyltransferase, which is the last enzyme in capsaicinoid biosynthesis. The encoded protein is called capsaicin synthase (CS) because vanillylamine from the phenylpropanoid pathway and the branched-chain fatty acid are condensed at the ends of two pathways. Stellari et al. (2010) discovered *Pun2*, a second locus affecting pungency in pepper, which was not allelic to the *Pun1* locus. However, the gene encoded by the *Pun2* locus is not known. The *Pun3* gene encodes the transcription factor CaMYB31, which plays a role as a master regulator of capsaicinoid biosynthetic genes in pepper including *Pun1* (Arce-Rodriguez and Ochoa-Alejo, 2017; Han et al., 2019; Zhu et al., 2019). The putative aminotransferase (*pAMT*) encodes a protein that catalyzes the formation of vanillylamine from vanillin in the phenylpropanoid pathway (Lang et al., 2009). Recently, a putative ketoacyl-ACP reductase (*CaKR1*) in the branched-chain fatty acid pathway was found to be related to pungency in *Capsicum chinense* (Koeda et al., 2019).

Capsaicinoid content is a quantitative trait in pepper that is regulated by multiple genes (Sanatombi and Sharma, 2008), and many studies have used QTL mapping to identify the genetic factors that regulate capsaicinoid content (Ben‐Chaim et al., 2006; Blum et al., 2003; Yarnes et al., 2013). The genes underlying the QTL often correspond to known structural genes (Han et al., 2018).

Recessive alleles of these genes explain the loss of pungency in *Capsicum* species. The first reported mutant allele of *Pun1* was identified with a large 2.5 kb deletion spanning 1.8 kb of the putative promoter and 0.7 kb of the truncated first exon (Stewart et al., 2005). This deletion was widespread throughout *Capsicum annuum* cultivars, especially in bell peppers. The second *Pun1* allele has a 4 bp deletion in the first exon region that creates an early stop codon in *C. chinense* (Stewart et al., 2007). In *Pun1^3^* reported in *Capsicum frutescens*, a premature stop codon leads to truncation in the second exon (Stellari et al., 2010). *Pun1^4^* was identified to have a single nucleotide insertion in the second exon, which generates a frameshift mutation (Kirii, et al., 2017). In non-pungent *Capsicum chacoense*, a recessive *pun2* allele is thought to be the reason for the non-pungency (Stellari et al., 2010). The mutant allele of *Pun3* was found in the non-pungent *C. annuum* accession with a nonsense mutation in the first exon, which leads to a premature stop codon (Han et al., 2019). More than 10 nonfunctional alleles of *pAMT* have been reported and were found to cause loss of pungency in *Capsicum* species from the results of Lang et al. (2009) that reported a nonsense mutation at *pAMT* in *C. annuum* ‘CH-19 Sweet.’ A mutation in *pAMT* alters capsaicinoids into capsinoids (Sutoh et al., 2006; Lang et al., 2009). Capsinoid biosynthesis is also regulated by *Pun1* (Han et al., 2013).

The *Pun2* locus has not been studied since it was first reported (Stellari et al., 2010). The *Pun2* locus was suggested as a novel locus of pungency based on the complementation tests of *Capsicum chacoense* PI260433-np with other known *Pun1* alleles, including *Pun1^1^*, *Pun1^2^*, *Pun1^3^*, and *Pun1^4^* (Stewart et al., 2005; Stewart et al., 2007; Stellari et al., 2010; Kirii et al., 2018). By bulk segregant analysis, the marker ‘Hpms1-172’ was identified and was evidence that the *Pun2* locus was located on the upper arm of chromosome 7 (Stellari et al., 2010).

In this study, we attempted to identify the gene encoded by the *Pun2* locus using two F_2_ populations. First, we performed QTL mapping in an interspecific F_2_ population derived from a cross between *C. annuum* and *C. chacoense* PI260433-np, which contains the recessive *pun2* allele. As a result, one major and one minor QTL that affected non-pungency capsaicinoid content were identified. A candidate gene in the major QTL was nominated and mapped in an intraspecific F_2_ population derived from a cross between the pungent and the non-pungent *C. chacoense* accessions. The candidate gene mapping revealed that the *Pun2* locus encodes *pAMT*.

## Materials and methods

### Plant materials and mapping population constructions

A non-pungent *Capsicum* species indigenous to South America, *C. chacoense* ‘PI260433-np,’ and a pungent Korean landrace *C. annuum* ‘Jeju’ were used as parental lines to construct an interspecific F_2_ population (PJ) for QTL analysis. PI260433-np is known to be non-pungent despite a functional allele of *Pun1* (Stellari et al., 2010). A total of 178 F_2_ plants were grown in the field of Seoul National University farm (Suwon, Republic of Korea) in 2019. The non-pungent PI260433-np was crossed with the pungent ‘PI260433-p’ to construct an intraspecific F_2_ population (PP). *C. chacoense* PI260433 is a wild pepper that shows a polymorphism in fruit pungency (Tewksbury et al., 2006; Tewksbury et al., 2008). A total of 113 F_2_ plants from a cross between PI260433-p and PI260433-np were grown in the greenhouse at Seoul National University (Suwon, Korea) from 2021 to 2022. An allelism test was carried out by crossing *C. chacoense* ‘PI260433-np’ with non-pungent cultivars *C. annuum* ‘YCM334’ and *C. annuum* ‘ECW30R.’ If two recessive genes are allelic, they will fail to complement each other in the F_1_ hybrids.

### Genomic DNA (gDNA) extraction

gDNA was extracted from young leaves of plant materials using a modified cetyltrimethylammonium bromide (CTAB) method (Han et al., 2018). Young leaf tissues were frozen in liquid nitrogen and finely ground using a 5 mm steel bead with a vortex mixer (DAIHAN Scientific, Wonju, Korea). The concentration and quality of extracted gDNA were measured with a Nanodrop spectrophotometer (BioTek, Winooski, VT, USA) and diluted to a final concentration of 20 ng/μL in triple distilled water (TDW).

### Phenotyping with Gibb’s screening and HPLC analysis

Mature green fruits harvested from each plant were sampled. Placenta tissues were used for Gibb’s analysis (Jeong et al., 2012). Placenta tissues were placed on filter paper and the same volume of 2,6-dichloroquinon-4-chloroimide (Gibb’s reagent; Sigma-Aldrich, Saint Louis, MI, USA) was sprayed. The filter paper with sprayed spots was steamed for 30 s with ammonia gas. If the color changed to blue, the samples were determined to be pungent. For HPLC analysis, at least three pepper fruits at the mature green (MG) stage were harvested, and the placental tissue was separated from each fruit. For the ‘PP’ F_2_ population, harvest was done in the same manner, but the whole fruits including seeds and the pericarp were for HPLC analysis. Pre-processed samples were analyzed by HPLC at the National Instrumentation Center for Environmental Management (NICEM; Seoul, Korea) to measure the capsaicinoid and capsinoid contents. Total capsaicinoids were calculated as the sum of capsaicin and dihydrocapsaicin content, and total capsinoids were calculated as the sum of capsiate and dihydrocapsiate contents.

### GBS library preparation and sequencing

Genomic DNA of ‘PJ’ F_2_ individuals and three replications of each parent were used to construct the Illumina sequencing library for GBS followed by Truong et al. (2012). Genomic DNAs were digested with *Eco*RI and *Mse*I, and then *Mse*I adapters and *Eco*RI adapters with different barcodes were used to ligate DNA fragments. Each sample with the same quantity of adapter-ligated DNA fragments was pooled for sequencing. Single-end sequencing was performed on one line of an Illumina Hiseq 2000 (Illumina, San Diego, CA, USA) at Macrogen Inc. (Seoul, Republic of Korea).

### SNP analysis

The adapter and barcodes were removed using CLC genomic workbench software version 8.0 (CLC Bio, Aarhus, Denmark). The reference genome of *C. annuum* ‘Dempsey’ (Lee et al., 2022 was used to align trimmed reads using Burrows-Wheeler Aligner version 0.7.12 (Li, 2013). To convert the alignment files into BAM files, Sequence Alignment/Map (SAM) tools version 1.1 was used. Picard Tools version 1.119 was then used to manipulate the SAM files and perform duplicate marking and sorting. The Genome Analysis Toolkit (GATK) UnifiedGenotyper version 3.3, with the criteria of a QUAL value larger than 30 and a minimum depth of 3, was used to further sort and filter SNPs.

### Bin map construction

The construction of a bin map was done using the modified sliding-window approach to reduce variant calling errors and the calculation of recombination breakpoints (Han et al., 2016). SNPs with non-polymorphic and missing data were removed. The ratio of SNPs with both parental genotypes was calculated for each window, defined as 25 linked SNPs, and the overall genotype of each window was decided. SNPs with ratios over 0.7 were defined as paternal and maternal genotypes; an SNP ratio between 0.3 and 0.7 was defined as heterozygous genotype. Construction of linkage maps was conducted with Carthagene software (De Givry et al., 2005). The criteria for the construction of a linkage map were a LOD score threshold of 4.0 and a maximum distance of 50 cM. The calculation of distance between bin markers was done with the Kosambi mapping function. The final linkage map was drawn using MapChart2.3 software (Voorips, 2002). The *C. annuum* ‘Dempsey’ reference genome was used to construct the bin map. The comparison with the physical location of bin markers was carried out using MapChart2.3 software.

### Genetic mapping of *Pun2*

A high-density genetic map from GBS data and phenotype data for capsaicin and dihydrocapsaicin contents in placenta tissue were used for QTL analysis. Composite interval mapping (CIM) was performed using Windows QTL cartographer v2.5 (Wang et al., 2012). The LOD threshold was determined using 1,000 permutations with a 5% probability for each chromosome and trait. The phenotypic variation proportion explained by each QTL was explained and estimated using the R^2^ (%) value.

### Sequence analysis and marker tests

PCR was performed using EzPCR™ XO 5x Master Mix (Elpis, Korea) with 100 ng of gDNA template and 0.5 μL of 10 pmol gene-specific primers for *Pun1*, *Pun3*, *CaKR1*, and *pAMT* in a total volume of 25 μL. The PCR conditions were as follows: one cycle of pre-denaturation at 95°C for 3 min and 25–28 cycles of 95°C for 30 s, 58°C for 30 s, and 72°C for 1 min. The resulting amplicons were separated on a 1% agarose gel. Sanger sequencing was performed at Macrogen (Seoul, Korea), and the nucleotide sequences were analyzed by Lasergene’s SeqMan program (DNASTAR, Madison, WI, USA). For genotyping of a novel *pamt* allele, a KASP marker set based on an indel in the 8th exon of *pAMT* was developed: 5’-(FAM)-GCTTCCTCCAATGAATCAAGCATCA-3’ (FAM-tailed forward primer), 5’-(HEX)-GCTTCCTCCAATGCATCAAAAATTGA-3’, and 5’- CCTGGCAAGTGATAGGCCCAATAA-3’ (common reverse primer). Thermocycling and endpoint genotyping for the KASP assays were performed in a Roche LC480 (Roche Applied Science, Indianapolis, IN, USA). The composition of the KASP mixture was as follows: 100 ng of plant DNA was mixed with 5 µL KASP V4.0 2X Mastermix (LGC Genomics, UK), 0.14 µL of the gene-specific KASP primer mix, and 0.06 µL MgCl_2_. The thermal cycling condition was 94°C hot-start for 15 min, followed by 10 cycles of 94°C for 20 s and 59°C for 60 s; the annealing temperature was decreased at a rate of 0.2°C/cycle, followed by 26 cycles at 94°C for 20 s and 57°C for 60 s and 3 cycles of 94°C for 20 s and 59°C for 60 s. The cooling cycle was at 37°C for 2 min, and the final fluorescent value was read at the same temperature as for endpoint genotyping.

## Results

### Characterization of parental lines

In HPLC analysis, the capsaicinoid content of Jeju was 5,417.5 2 μg·gDW^-1^ in placenta tissue and capsinoid content was 482.2 μg·gDW^-1^. The pungent PI260433-p contained 33,027 μg·gDW^-1^ of capsaicinoids, whereas the non-pungent line PI260433-np contained 139 μg·gDW^-1^ of capsaicinoids (**Table 1**). In contrast, the total capsinoid content in PI260433-np was higher than the content in PI260433-p: the total capsinoid content in PI260433-p and PI260433-np was 1,464 ± 512 μg·gDW^-1^ and 7,898 ± 686 μg·gDW^-1^, respectively. If *Pun1* or *Pun3* was not functioning in PI260433-np, which might result in no capsaincinoid accumulation, there would be no capsinoid biosynthesis either. These data indicate that the low pungency in PI260433-np might be related to a mutation of *pAMT*.

**Table 1:** Levels of capsaicinoids and capsinoids in the placental tissue in parental lines.

Line	Pungency	Capsaicinoid content(μg·gDW^-1^)	Capsinoid content(μg·gDW^-1^)
Jeju	Pungent	5,417	482
PI260433-p	Pungent	33,027 ± 10,394	1,464 ± 512
PI260433-np	Non-pungent	139 ± 14	7,898 ± 686

### Phenotyping and genotyping of an interspecific population

**Figure 1:**
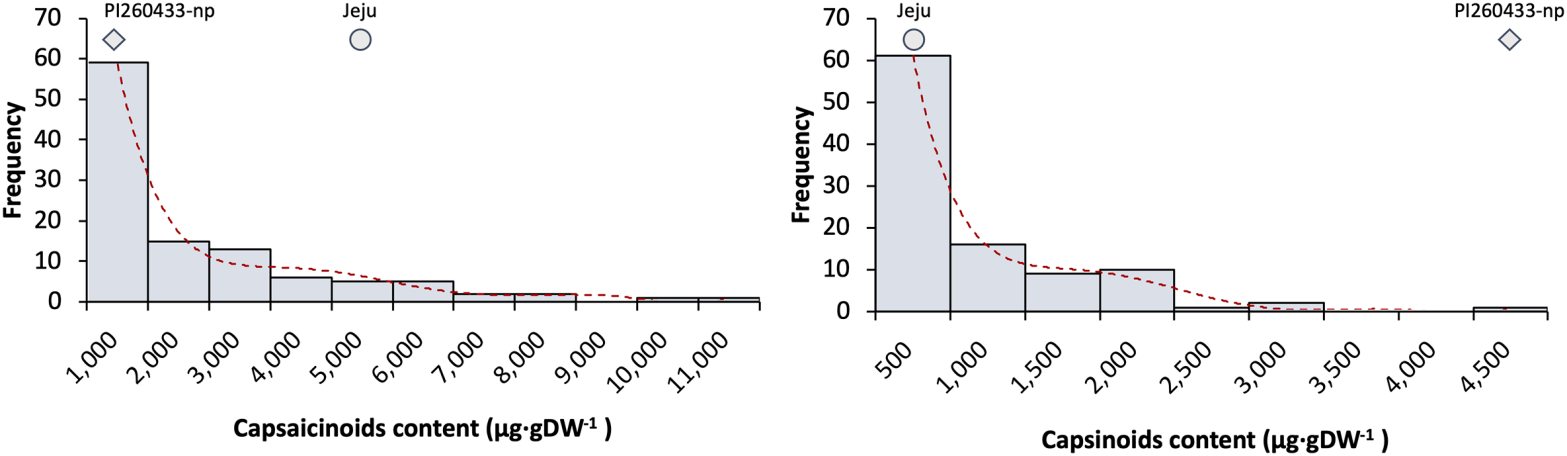
Frequency distribution of capsaicinoid and capsinoid contents in the ‘PJ’ F2 population. The x-axis indicates the number of plants (frequency), and the y-axis indicates the range of capsaicinoid (left) or capsinoid (right) amounts.

For evaluation of the pungency in the ‘PJ’ F_2_ population, Gibb’s analysis was first conducted at least two times using mature green stage fruits. If the color of samples turned blue, it was determined to be pungent, while samples with no color changes were determined to be non-pungent. Of 171 F_2_ samples, 116 were pungent, and 55 samples were non-pungent. The segregation ratio of non-pungent vs. pungent peppers in the F_2_ population was 2.11:1 (**Table S1**). For more accurate phenotype analysis, HPLC analysis of 120 F_2_ plants was conducted to confirm the Gibb’s analysis results. The mean capsaicinoid content of the placenta tissue of the F_2_ population was 1,561 ± 193 μg·gDW^-1^and the mean capsinoid content was 534 ± 64 μg·gDW^-1^ (**Table 2**). Plants with a capsaicinoid content more than 100 μg·gDW^-^ were considered pungent, whereas plants with a content less than 100 μg·gDW^-^ were considered non-pungent. The segregation ratio in phenotype by HPLC analysis was 1.72:1 (**Table S1**). The distribution of both capsaicinoid and capsinoid content exhibited a positive skew; the positive skew implies the mean is greater than the median (**Figure 1**).

**Table 2:** The total capsaicinoid and capsinoid contents in the ‘PJ’ F_2_ population.

Phenotype^*^	Number of plants	Capsaicinoid content (μg·gDW^-1^)	Capsinoid content (μg·gDW^-1^)
Pungent (>100 μg·gDW^-1^)	76	2,446 ± 254	525 ± 71
Non-pungent (< 100 μg·gDW^-1^)	44	32 ± 4	548 ± 126

^*^The phenotype was determined by the content of capsaicinoids.

Genotyping of the ‘PJ’ F_2_ population was carried out with GBS. *EcoR1/Mse1*-digested DNA was used for the construction of GBS libraries. The average number of reads per sample was 2,803,611, and the reads were aligned to the *C. annuum* ‘Dempsey’ reference genome (Lee et al., 2022). A total of 10,136 SNPs was obtained from GBS analysis (**Table S2**). The density of SNPs showed differences among the chromosomes (**Figure S1**).

To construct a linkage map, the modified sliding window approach was used to correct missing data and genotyping errors (Huang et al., 2009; Chen et al., 2014; Han et al., 2016). To determine recombination breakpoints, 25 consecutive SNPs were considered as one sliding window. CarthaGene software was used to construct a genetic linkage map. The map consisted of 1,422 bins with an average genetic distance of 3.86 cM. Among the 12 linkage groups, the genetic distance of chromosome 4 was the longest at 752 cM, and the distance of chromosome 2 was the shortest at 234.1 cM (**Table S3**). The bin map developed using 1,422 bin markers was aligned to the *C. annuum* ‘Dempsey’ reference genome to compare the physical position of the bins (**Figure S2**).

### QTLs associated with capsaicinoid content

**Figure 2:**
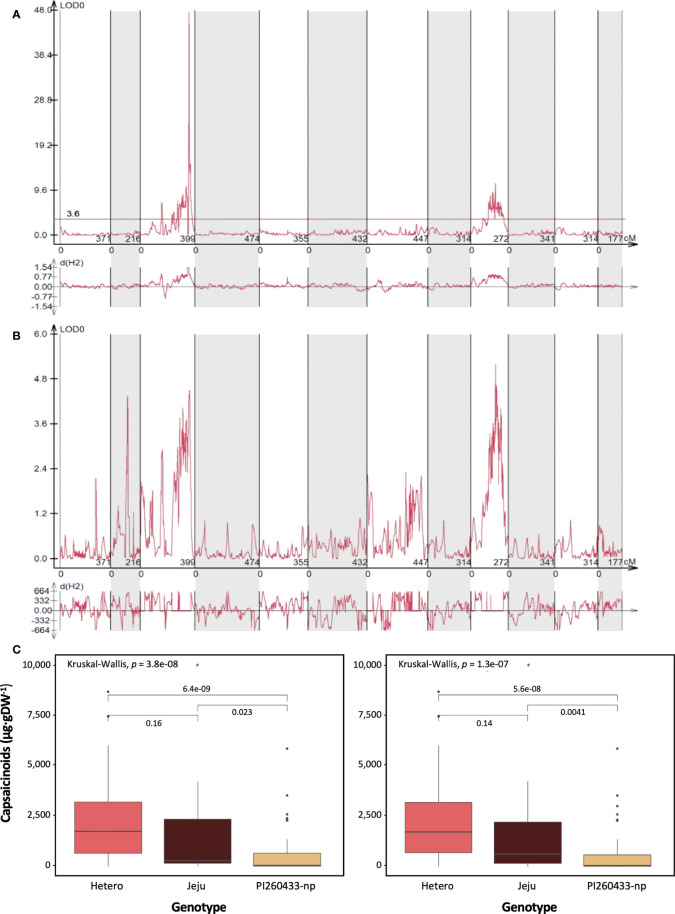
QTL mapping of capsaicinoid content in the ‘PJ’ F_2_ population. **(A)** The detected QTLs were associated with the phenotypic data from Gibb’s analysis. The most dominant QTL was located on chromosome 3, and the other QTL was detected on chromosome 9. **(B)** The detected QTLs were associated with the phenotypic data from HPLC analysis. No significant QTLs were detected. **(C)** Comparison of QTLs and SNPs associated with capsaicinoid content and box plots of capsaicinoid content regulated by two markers representing the two QTLs. Bin markers used for box plot were Bin7_25_1M_249000000 (left) and Bin10_25_1M_204000000 (right) in the ‘PJ’ F_2_ population.

QTLs controlling the total capsaicinoid content were detected in the ‘PJ’ F_2_ population. Two phenotype data sets and a high-density bin map were used to identify genetic factors. The QTLs associated with the pungency level by Gibbs’s analysis were mainly located on chromosomes 3 and 9, as shown in **Figure 2A**. A total of 13 and 14 QTLs for the capsaicinoid contents were detected on chromosomes 3 and 9, respectively (**Table 3**). Of 27 QTLs, 18 showed negative additive effects. Among all detected QTLs, *Pun2_PJ_Gibbs_3.11* on chromosome 3 showed the highest LOD and R^2^ value, explaining 69% of the total phenotypic variations in the ‘PJ’ F_2_ population. To compare the physical location of QTLs, the *C. annuum* ‘Dempsey’ reference genome (Lee et al., 2022) was used. By comparing the physical positions of the detected QTLs on chromosomes 3 and 9 with the copies of the *pAMT* gene, we found that the major QTL *Pun2_PJ_Gibbs_3.11* was located adjacent to the position of the copy of the *pAMT* gene on chromosome 3 (**Figure S3**). To validate the detected QTLs, phenotype data from HPLC analysis were used. Although several relatively high peaks were observed on three chromosomes including 3 and 9, no significant QTLs were detected, as was detected by Gibbs’s analysis (**Figure 2B**).

**Table 3:** Quantitative trait loci (QTL) associated with the pungency level measured by Gibbs’s analysis of the placental tissues in the ‘PJ’ F_2_ population.

QTL	Chromosome	Position (cM)	LOD	R^2^	Additive	Dominant	Location (cM)
*Pun2_PJ_Gibbs_3.1*	3	153.91	7.09	0.18	-0.87	0.64	151.4-157.8
*Pun2_PJ_Gibbs_3.2*	3	242.11	5.07	0.22	-1.15	0.98	237.4-248.3
*Pun2_PJ_Gibbs_3.3*	3	269.71	7.49	0.15	-0.01	0.93	269.1-272
*Pun2_PJ_Gibbs_3.4*	3	285.21	6.83	0.14	0.95	0.7	280.5-288.8
*Pun2_PJ_Gibbs_3.5*	3	297.21	7.05	0.18	-0.85	1	295.2-301.4
*Pun2_PJ_Gibbs_3.6*	3	303.31	8.47	0.18	-0.07	1.04	303.1-306.6
*Pun2_PJ_Gibbs_3.7*	3	310.81	8.81	0.19	-0.68	1	308.9-311.6
*Pun2_PJ_Gibbs_3.8*	3	325.61	7.43	0.15	0.07	0.94	318.2-328.5
*Pun2_PJ_Gibbs_3.9*	3	333.51	10.47	0.21	-0.92	1.05	333.2-335.7
*Pun2_PJ_Gibbs_3.10*	3	343.21	7.87	0.16	-0.33	0.91	342-343.8
***Pun2_PJ_Gibbs_3.11***	**3**	**352.81**	**47.59**	**0.69**	**0.94**	**1.06**	**351.8-354.1**
*Pun2_PJ_Gibbs_3.12*	3	360.31	15.45	0.34	0.56	0.88	359-360.4
*Pun2_PJ_Gibbs_9.1*	9	129.11	7.29	0.15	0.27	0.94	127.4-130.5
*Pun2_PJ_Gibbs_9.2*	9	138.91	8.58	0.17	0.11	0.98	137.9-139.9
*Pun2_PJ_Gibbs_9.3*	9	148.41	6.07	0.13	-0.35	0.89	144.3-149.8
*Pun2_PJ_Gibbs_9.4*	9	156.31	9.26	0.21	-1.13	1.02	153.4-158.8
*Pun2_PJ_Gibbs_9.5*	9	161.41	6.87	0.16	-0.59	0.95	159.4-162.3
*Pun2_PJ_Gibbs_9.6*	9	169.71	7.76	0.16	-0.26	0.98	168.6-171.4
*Pun2_PJ_Gibbs_9.7*	9	175.91	11.17	0.27	1.16	0.89	174.4-177.9
*Pun2_PJ_Gibbs_9.8*	9	187.11	6.06	0.16	-0.21	0.96	182.1-190.1
*Pun2_PJ_Gibbs_9.9*	9	193.41	7.19	0.15	-0.44	0.86	190.9-194.3
*Pun2_PJ_Gibbs_9.10*	9	201.41	7.2	0.17	-0.21	1.01	200.3-203.4
*Pun2_PJ_Gibbs_9.11*	9	207.81	6.36	0.13	0.06	0.89	205.9-214.9
*Pun2_PJ_Gibbs_9.12*	9	216.71	7.09	0.15	0	0.92	214.9-217.6

Bold values indicate the most dominant QTL with the highest LOD value among all detected QTLs.

To validate the effect of the QTLs, individual plants in the ‘PJ’ F_2_ population were sorted into three groups according to genotype (‘PI260433-np’, ‘Jeju,’ or heterozygote) at bin markers located within the QTLs. The box plot was drawn with Bin7_25_1M_249000000 for the QTL detected on chromosome 3 and Bin10_25_1M_204000000 for the QTL detected on chromosome 9. All markers on both chromosomes were highly associated with the capsaicinoid content (**Figure 2C**). This result indicated that the QTLs of the pungency level measured by Gibb’s analysis correlated with the actual capsaicinoid content.

### Identification of nucleotide sequence variations in *Pun1*, *Pun3*, *CaKR1*, and *pAMT*

**Figure 3:**
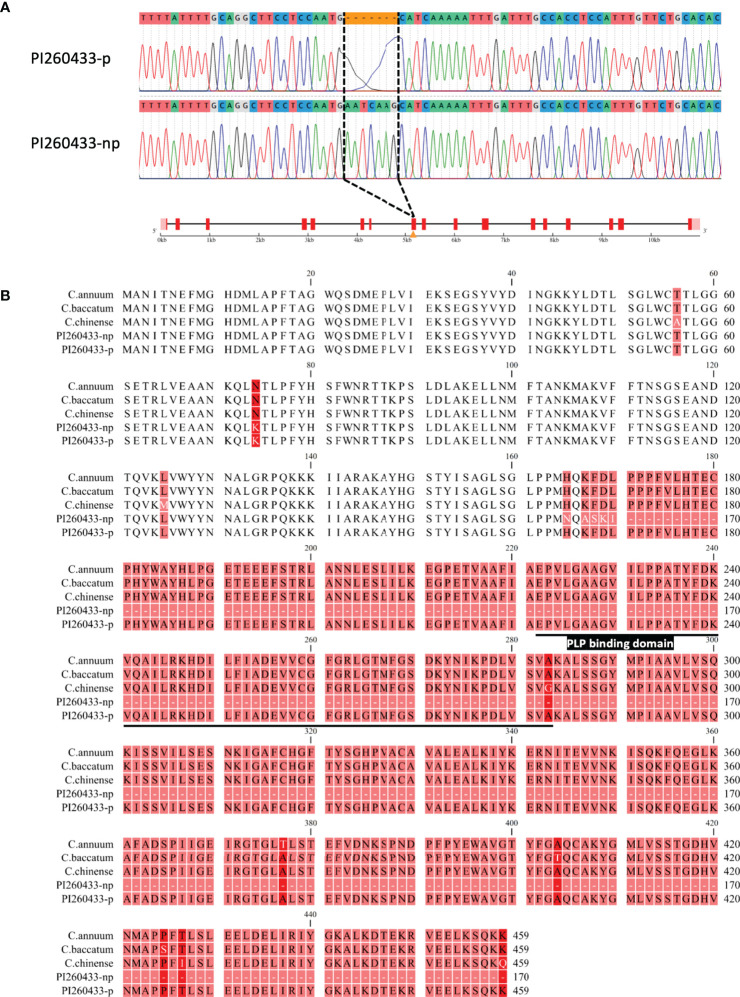
Schematic structure of *pamt^11^*. **(A)** Gene structure of *pAMT*. A sequence of 7 bp (5’-AATCAAG-3’) is inserted in the 8^th^ exon of *pAMT* in PI260433-np. Boxes and lines indicate exons and introns, respectively. **(B)** Amino acid sequences of translated proteins of *pAMT*. The pyridoxal 5-phosphate (PLP) binding domain is underlined.

To confirm whether *pAMT*, which is located at the major QTL position on chromosome 3, contributes to the difference in pungency of *C. chacoense*, we investigated the genetic variations of the *pAMT* gene between the two *C. chacoense* accessions, PI260433-p and PI260433-np. The full-length sequences of *pAMT* were obtained by Sanger sequencing and compared, and a 7 bp insertion (5’-AATCAAG-3’) in the 8th exon of *pAMT* was found in PI260433-np but not in PI260433-p (**Figure 3A**). This insertion caused an early stop codon, resulting in a truncated translation product lacking the pyridoxal 5-phosphate (PLP) binding domain vital for *pAMT* enzymatic activity (**Figure 3B**). This mutation has never been reported in *Capsicum* spp. and was named *pamt^11^*. There were no variations in the gDNA sequence between PI260433-p and PI260433-np in other structural genes including *Pun1*, *Pun3*, and *CaKR1* except for several SNPs and InDels that altered some amino acid sequences (**Figures S4**–**S6**). Therefore, the difference in fruit pungency between PI260433-np and PI260433-p may due to the mutation of *pAMT*.

### Co-segregation of a novel *pamt* allele with capsinoid contents in an F_2_ population

**Figure 4:**
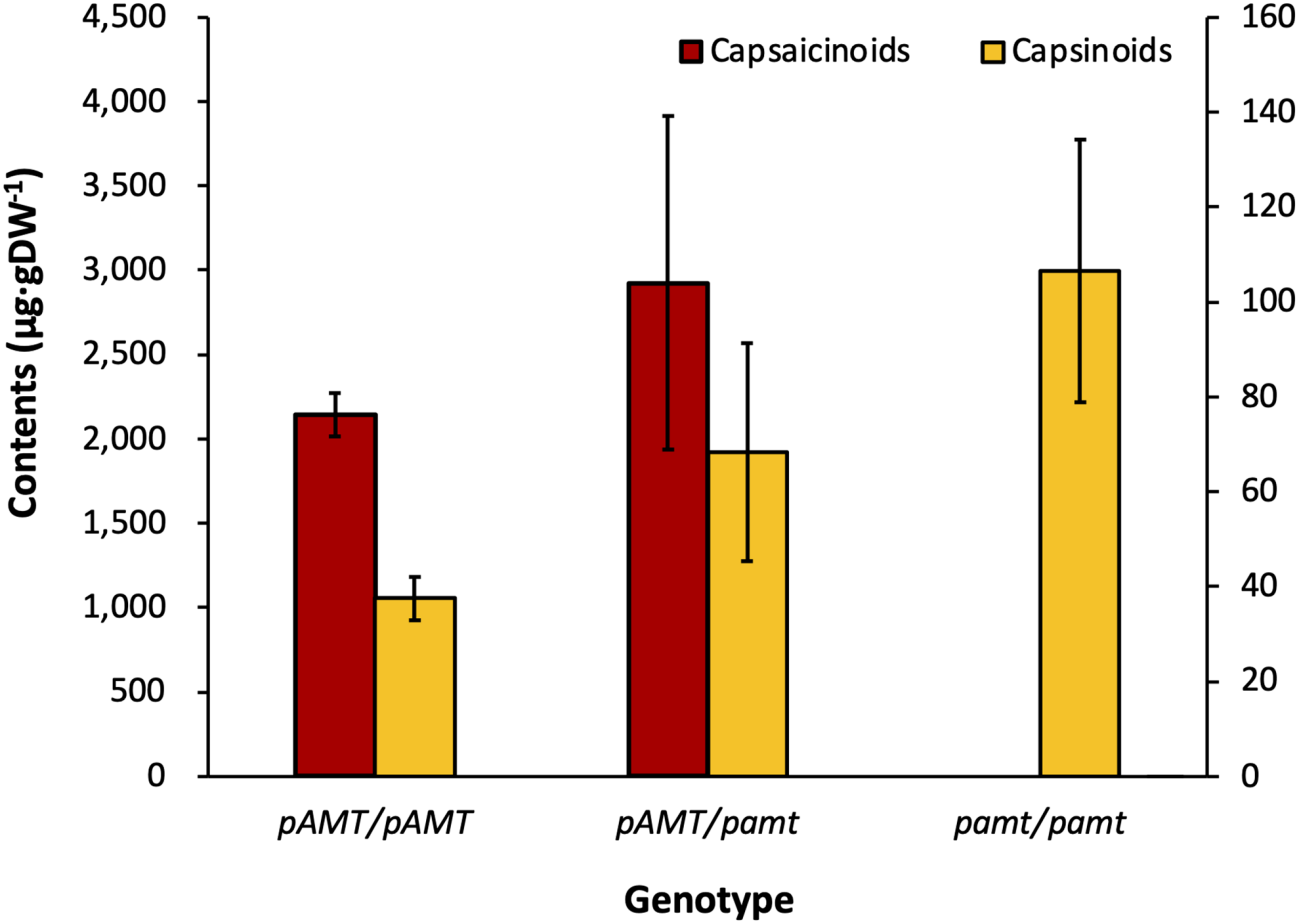
Capsaicinoid and capsinoid contents by genotypes of *pAMT* in the ‘PP’ F_2_ population. The capsaicinoid content was not able to be determined in the group of homozygous *pamt^11^*, whereas the capsinoid content was the largest among all genotype groups.

A KASP marker based on the novel 7 bp indel of *pAMT* was designed to test the association of this mutation and pungency in an F_2_ mapping population obtained from a cross between PI260433-p and PI260433-np. A total of 113 F_2_ plants was genotyped by KASP assay. The F_2_ population was segregated into three genotypes, two homozygous genotypes (*pAMT^P^*/*pAMT^P^*, *pAMT^NP^/pAMT^NP^*), and one heterozygous genotype (*pAMT^P^*/*pAMT^NP^*). The segregation ratio of three genotypes, *pAMT^P^*/*pAMT^P^*, *pAMT^P^*/*pAMT^NP^*, and *pAMT^NP^/pAMT^NP^*, in the F_2_ population was 43:41:29 (or 1.48:1.41:1). The HPLC analysis of the ‘PP’ F_2_ population showed that the segregation ratio of phenotype was 2.88:1 (pungent: non-pungent) with a *p*-value of 0.86 (>0.05) (**Table 4**). Comparison between thepungency phenotype and the genotype of *pAMT* confirmed that the capsaicinoid and capsinoid contents co-segregated with *pamt* ^11^ (**Figure 4**). Taken together, these results suggest that the non-pungency of *C. chacoense* ‘PI260433-np’ is due to *pamt^11^*, and *Pun2* encodes the *pAMT* gene.

**Table 4:** Segregation ratio of pungency phenotype in the ‘PP’ F2 population.

Population	Pungent : Non-pungent	Ratio	χ^2^ (3:1)^*^	*P*
PP F_2_	75:26	2.88:1	0.0297	0.86 > 0.05

^*^Significant limit of χ^2^ (P=0.05, df=1) = 3.84.

## Discussion

The pungency of pepper fruit is controlled by single genes and QTLs. The *pAMT* gene was proposed to encode a protein that catalyzes vanillin to vanillylamine in the capsaicinoid biosynthesis pathway (Curry et al., 1999; Blum et al., 2003; Abraham-Juárez et al., 2008). A mutation of *pAMT* leads to a loss of pungency with the production of capsinoids instead of capsaicinoids (Sutoh et al., 2006; Lang et al., 2009). The loss of the pungency gene *pun2* was previously reported in *C. chacoense* ‘PI260433-np’ (Stellari et al., 2010). In this study, we found that PI260433-np contained approximately 200 times lower levels of capsaicinoids and approximately 5 times higher levels of capsinoids than PI260433-p. PI260433-np did not result in a complete loss of pungency, as a small amount of capsaicinoids was detected, demonstrating that *Pun1* in PI260433-np encodes a functional protein. *pAMT* mutants produce a trace amount of capsaicinoids, whereas *Pun1* mutants completely lose pungency (Han et al., 2013; Tanaka et al., 2015). However, PI260433-np produced a notable amount of capsaicinoids, which indicated a malfunctioning *pAMT* gene.

To locate the *Pun2* locus, we first performed QTL mapping using an interspecific population derived from a cross between the non-pungent *C. chacoense* ‘PI260433-np’ and pungent *C. annuum* ‘Jeju,’ and two major QTLs were detected on chromosomes 3 and 9, where homologs of *pAMT* are located. One major QTL on chromosome 3 was located at the exact position of one of the *pAMT* copies. On chromosome 9, there was another copy of *pAMT*, but the position did not exactly match the QTL region. We postulated that the *pAMT* homolog on chromosome 3 might be the *Pun2* gene. To test this hypothesis, a candidate analysis using *pAMT* was performed, and the results identified a 7 bp insertion at the 8th exon in *pAMT* of ‘PI260433-np.’ This insertion causes a frameshift mutation leading to early termination of *pAMT* translation. This *pAMT* allele, named *pamt^11^*, co-segregated with the non-pungency in the F_2_ population derived from a cross between PI260433-p and PI260433-np. In this population, the non-pungency phenotype was segregated as a single gene rather than a quantitative trait, as was observed in the interspecific population. Interestingly, the capsaicinoid content was higher in heterozygous genotype of *pAMT*/*pamt* than in homozygous *pAMT*/*pAMT*. This may be due to the heterotic effect in intraspecific population (Naves et al., 2022). To date, several copies of *pAMT* have been reported: two on chromosome 3, one on chromosome 7, and one on chromosome 9 (Blum et al., 2003; Mazourek et al., 2009; Scossa et al., 2019). The positions of *pAMT* copies are frequently associated QTLs for capsaicinoid content. Ben-Chaim et al., 2006) reported two QTLs associated with the capsaicinoid content on chromosome 7 in an interspecific population derived from *C. annuum* and *C. frutescens* (Ben-Chaim et al., 2006). The position of one of two QTLs, *cap7.2*, was near *pAMT* on chromosome 7. Han et al. (2018) also reported that one of the QTLs for capsaicinoid content corresponded to the *pAMT* gene on chromosome 3. These results demonstrate that the *pAMT* genes on different chromosomes affect capsaicinoid biosynthesis.

We noted that the *Pun2* position reported in our study is different from previous reports. Stellari et al. (2010) reported that *Pun2* was located on the upper arm of chromosome 7; however, they were unable to show the exact position of the gene because the lack of polymorphic markers around the gene hindered mapping.

No nonfunctional *pAMT* gene in *C. chacoense* has been reported. Three and one *pAMT* mutant alleles were reported in *C. annuum* and *C. frutescens*, respectively (Lang et al., 2009; Tanaka et al., 2010a; Park et al., 2015; Tsurumaki and Sasanuma, 2019). Six *pAMT* mutant alleles were found in *C. chinense* (Tanaka et al., 2010b; Koeda et al., 2014; Tanaka et al., 2015; Tanaka et al., 2018). As *C. chacoense* is a wild *Capsicum* species, this species may have untapped traits that might be lost during the domestication of other *Capsicum* species (Mongkolporn and Taylor, 2011). The nonfunctional *pAMT* allele may be a valuable genetic resource for the breeding of high capsinoids cultivars.

In summary, we discovered a novel non-functional *pAMT* allele and identified the *Pun2* locus, which may encode the *pAMT* gene. This novel allele may be used as a breeding material to produce capsinoids in chili peppers.

## Data availability statement

The data presented in the study are deposited in the NABIC repository (https://nabic.rda.go.kr/nolog/NV-0748-000001/snpVcfView.do), accession number NV-0748.

## Author contributions

SY and D-GL contributed equally as the first authors of this work. SY wrote the manuscript and carried out the phenotyping and genotyping in the intraspecific population, the development of the molecular marker set, and data analysis. At the same time, D-GL conducted QTL mapping in the interspecific population. SB contributed to phenotyping, data analysis, and the development of figure sets. J-PH and SJ contributed to data processing and analysis. B-CK supervised the overall processes and revised the manuscript. All authors contributed to the article and approved the submitted version.

## Acknowledgments

This work was supported by the National Research Foundation of Korea (NRF) grant funded by the Korea government (MSIT) (No. 2021R1A2C2007472). This work was supported by a grant from the ‘New Breeding Technologies Development Program (Project No. PJ0165432022)’, Rural Development Administration, Republic of Korea.

## Conflict of interest

The authors declare that the research was conducted in the absence of any commercial or financial relationships that could be construed as a potential conflict of interest.

## Publisher’s note

All claims expressed in this article are solely those of the authors and do not necessarily represent those of their affiliated organizations, or those of the publisher, the editors and the reviewers. Any product that may be evaluated in this article, or claim that may be made by its manufacturer, is not guaranteed or endorsed by the publisher.

## Supplementary material

Supplementary Figure 1 SNP densities of the ‘PJ’ F2 population. The scale bar on the right indicates the number of SNPs within a 1 Mb window size.

Supplementary Figure 2 Comparison of the genetic map of the ‘PJ’ F_2_ population with the physical map. The scale bar on the left shows the genetic map position (cM).

Supplementary Figure 3 The comparison between the detected QTLs and the *pAMT* genes on chromosomes 3 and 9. The yellow box and the red arrow indicate the location of the QTL and the *pAMT* gene, respectively.

Supplementary Figure 4 Amino acid sequence of the translated protein of *Pun1*. Amino acid sequences were obtained from the reference genome of *C. annuum* ‘Takanotsume,’ *C. baccatum* ‘PBC81,’ and *C. chinense* ‘Habanero.’ The different residues are marked in white text in black boxes.

Supplementary Figure 5 Amino acid sequence of the translated protein of *Pun3*. Amino acid sequences were obtained from the reference genomes of *C. annuum* ‘Takanotsume’ and *C. baccatum* ‘PBC81.’ The different residues are marked in white text in black boxes.

Supplementary Figure 6 Amino acid sequences of the translated protein of *CaKR1*. Amino acid sequences were obtained from the reference genomes of *C. annuum* ‘Takanotsume,’ *C. baccatum* ‘PBC81,’ and *C. chinense* ‘Habanero.’ The different residues are marked in white text in black boxes.
