# Supplementary figures and images for: Genetic mapping revealed that the Pun2 gene in Capsicum chacoense encodes a putative aminotransferase
Source: Front Plant Sci. 2022 Nov 1;13:1039393. doi: 10.3389/fpls.2022.1039393 (PMC9664168; doi:10.3389/fpls.2022.1039393)

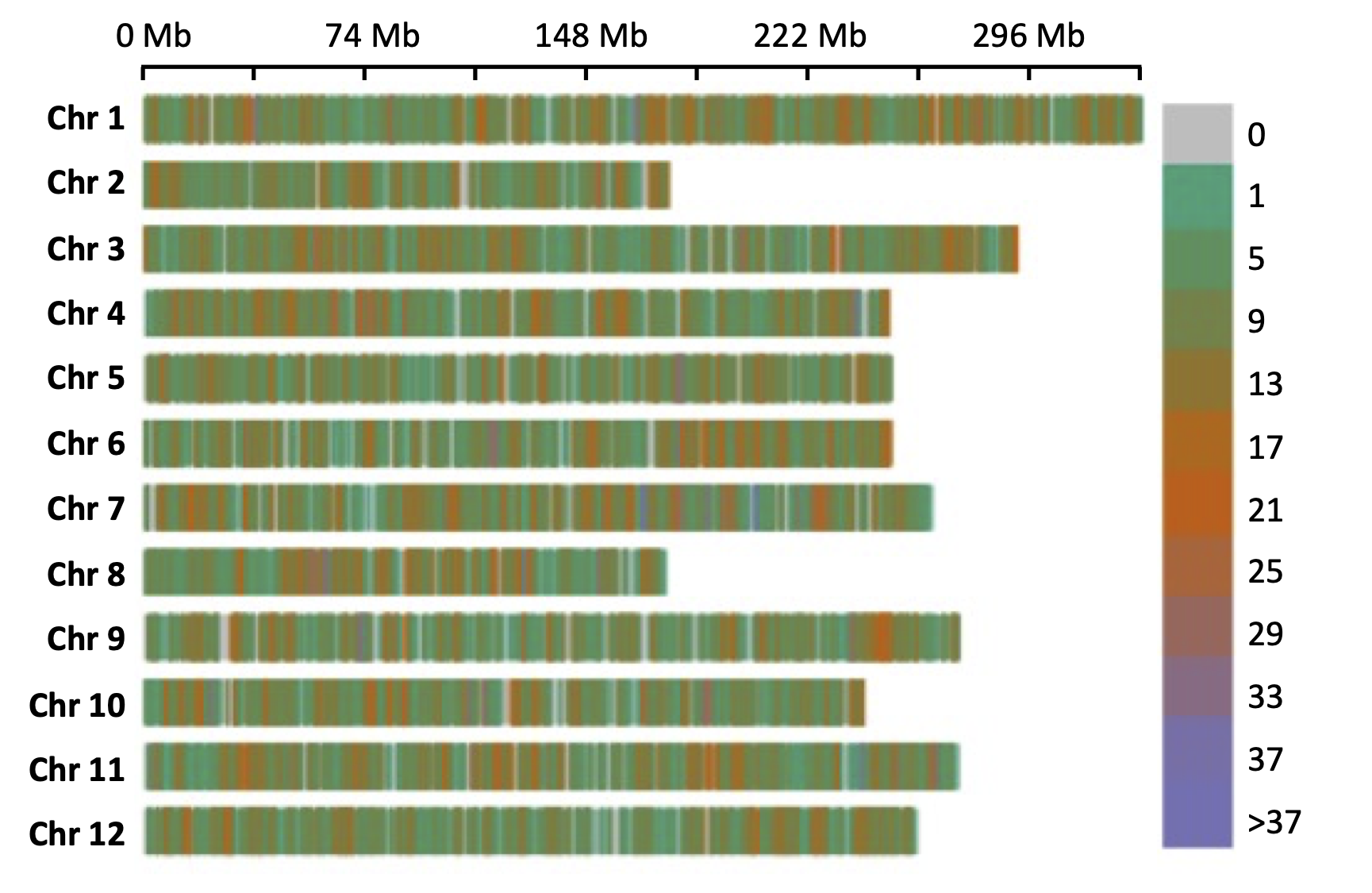

Supplement: Supplementary Figure 1 — SNP densities of the ‘PJ’ F2 population. The scale bar on the right indicates the number of SNPs within a 1 Mb window size. [file Image_1.tiff]

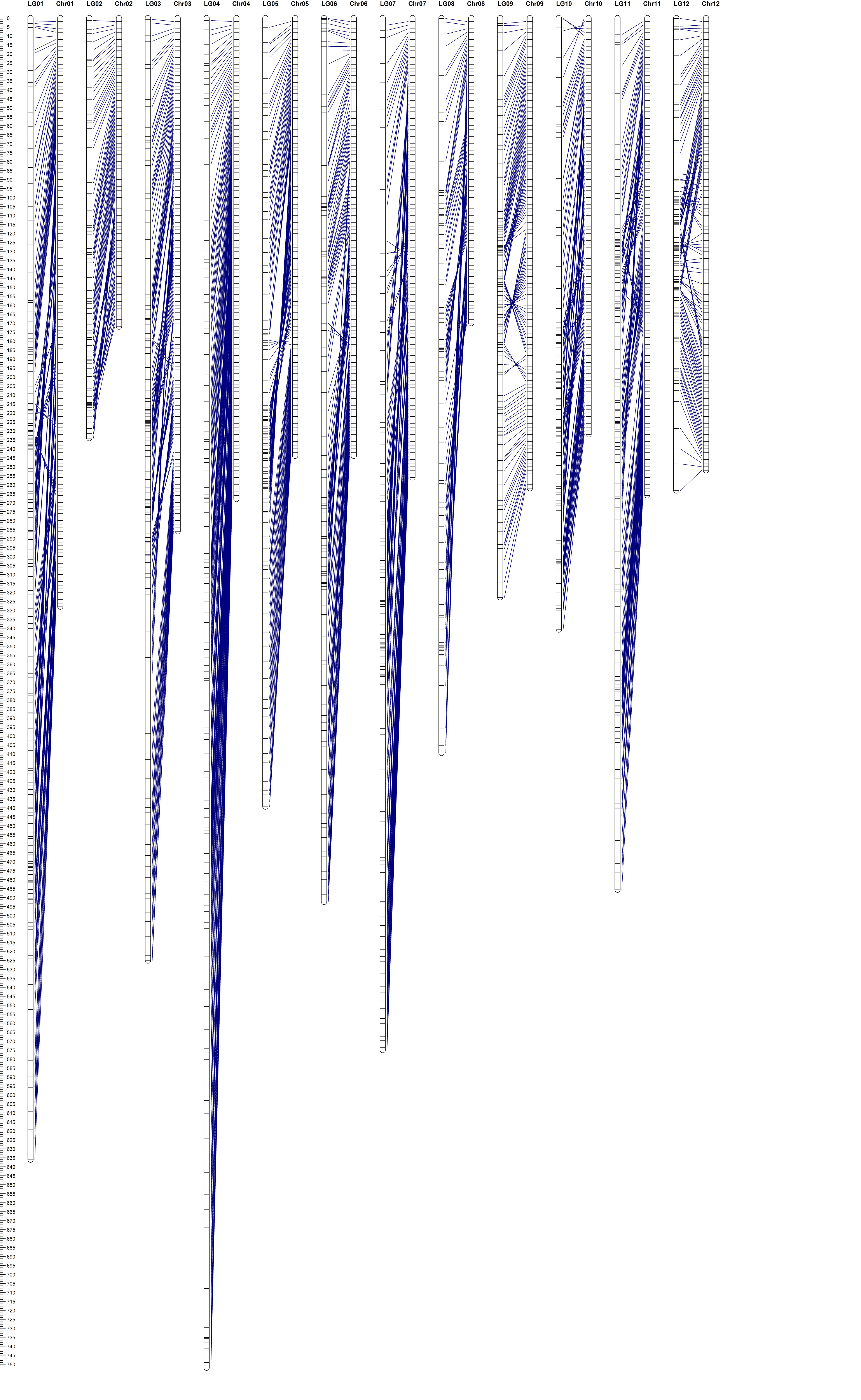

Supplement: Supplementary Figure 2 — Comparison of the genetic map of the ‘PJ’ F2 population with the physical map. The scale bar on the left shows the genetic map position (cM). [file Image_2.jpeg]

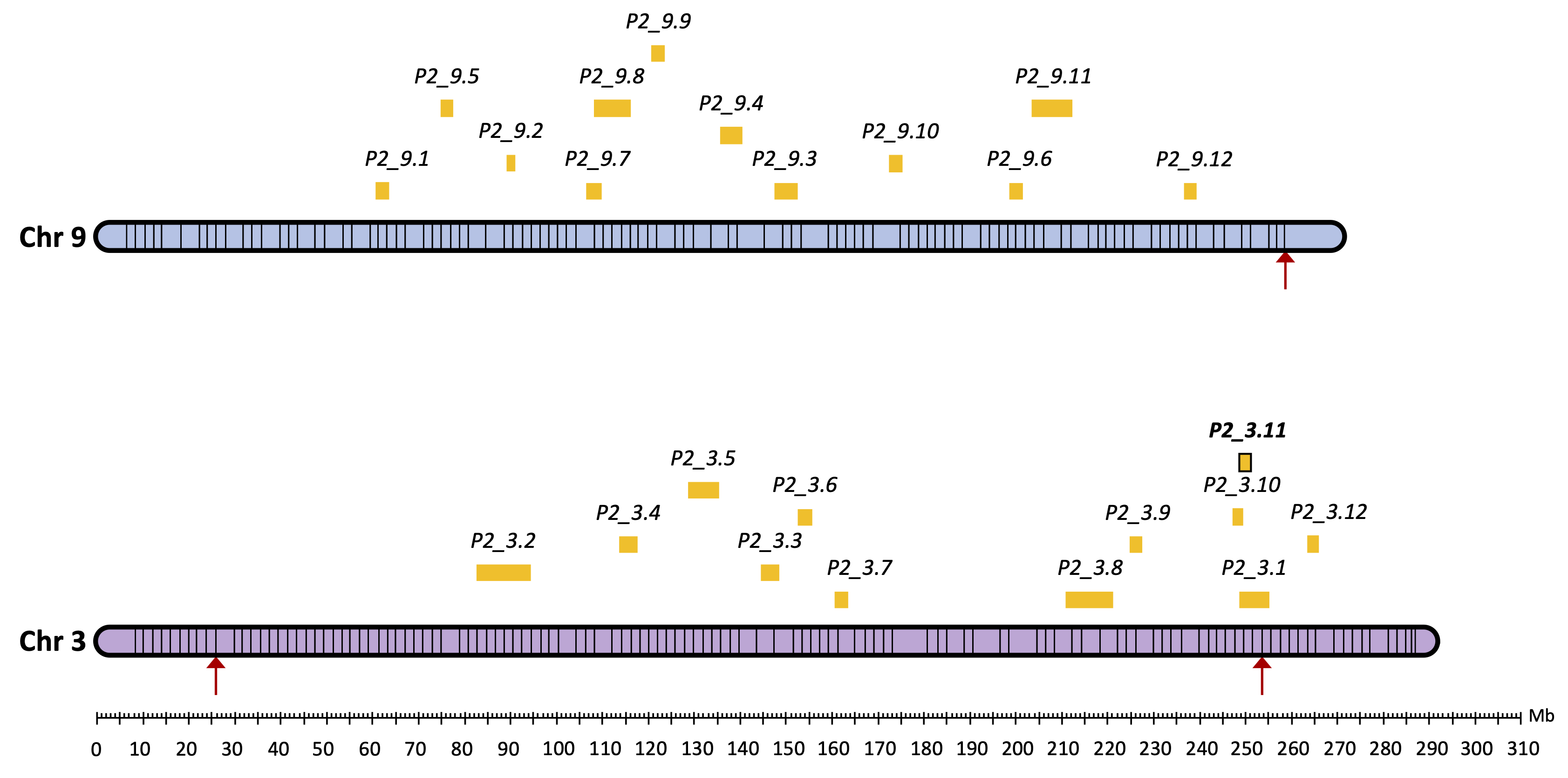

Supplement: Supplementary Figure 3 — The comparison between the detected QTLs and the pAMT genes on chromosomes 3 and 9. The yellow box and the red arrow indicate the location of the QTL and the pAMT gene, respectively. [file Image_3.jpg]

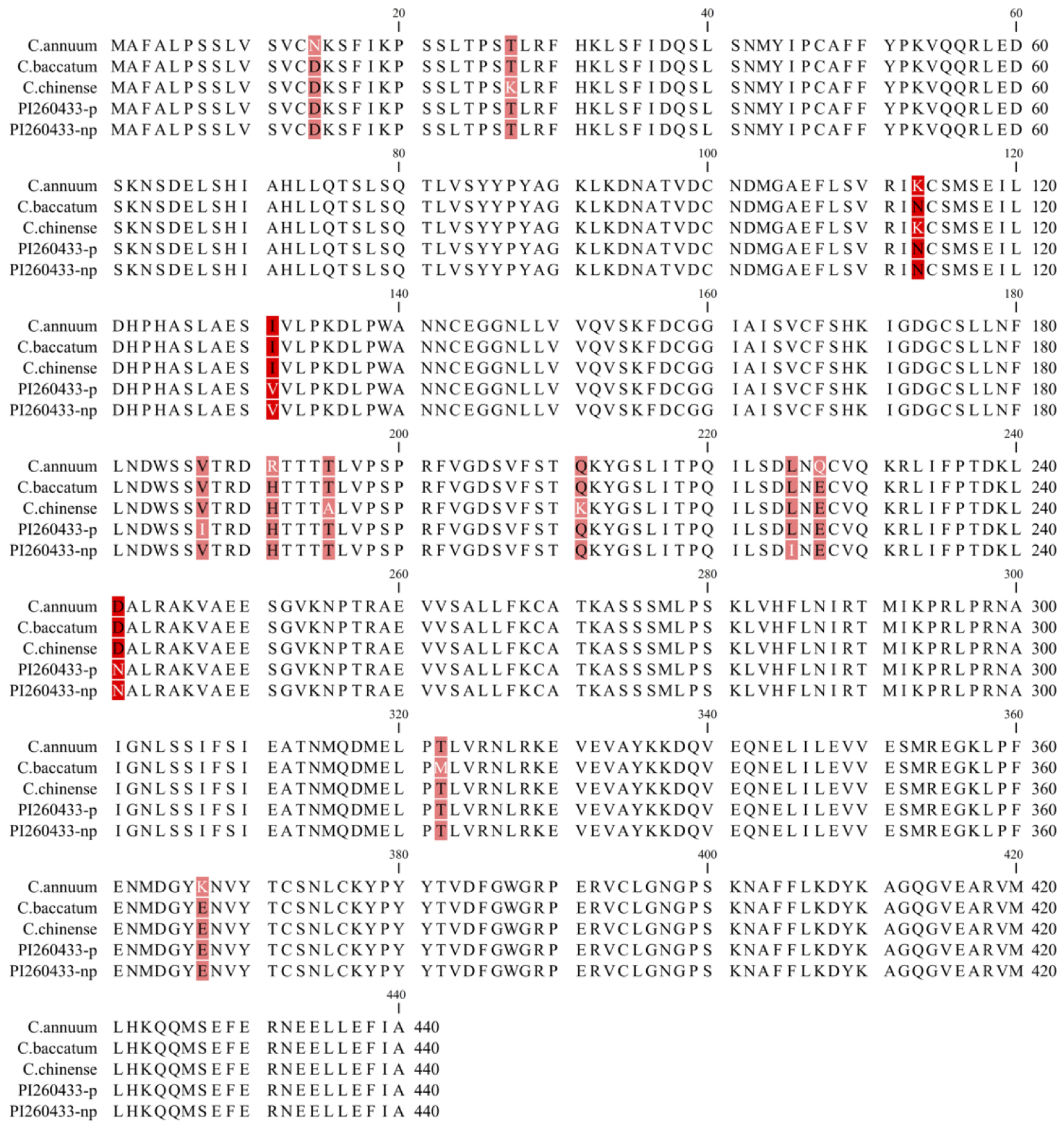

Supplement: Supplementary Figure 4 — Amino acid sequence of the translated protein of Pun1. Amino acid sequences were obtained from the reference genome of C. annuum ‘Takanotsume,’ C. baccatum ‘PBC81,’ and C. chinense ‘Habanero.’ The different residues are marked in white text in black boxes. [file Image_4.tiff]

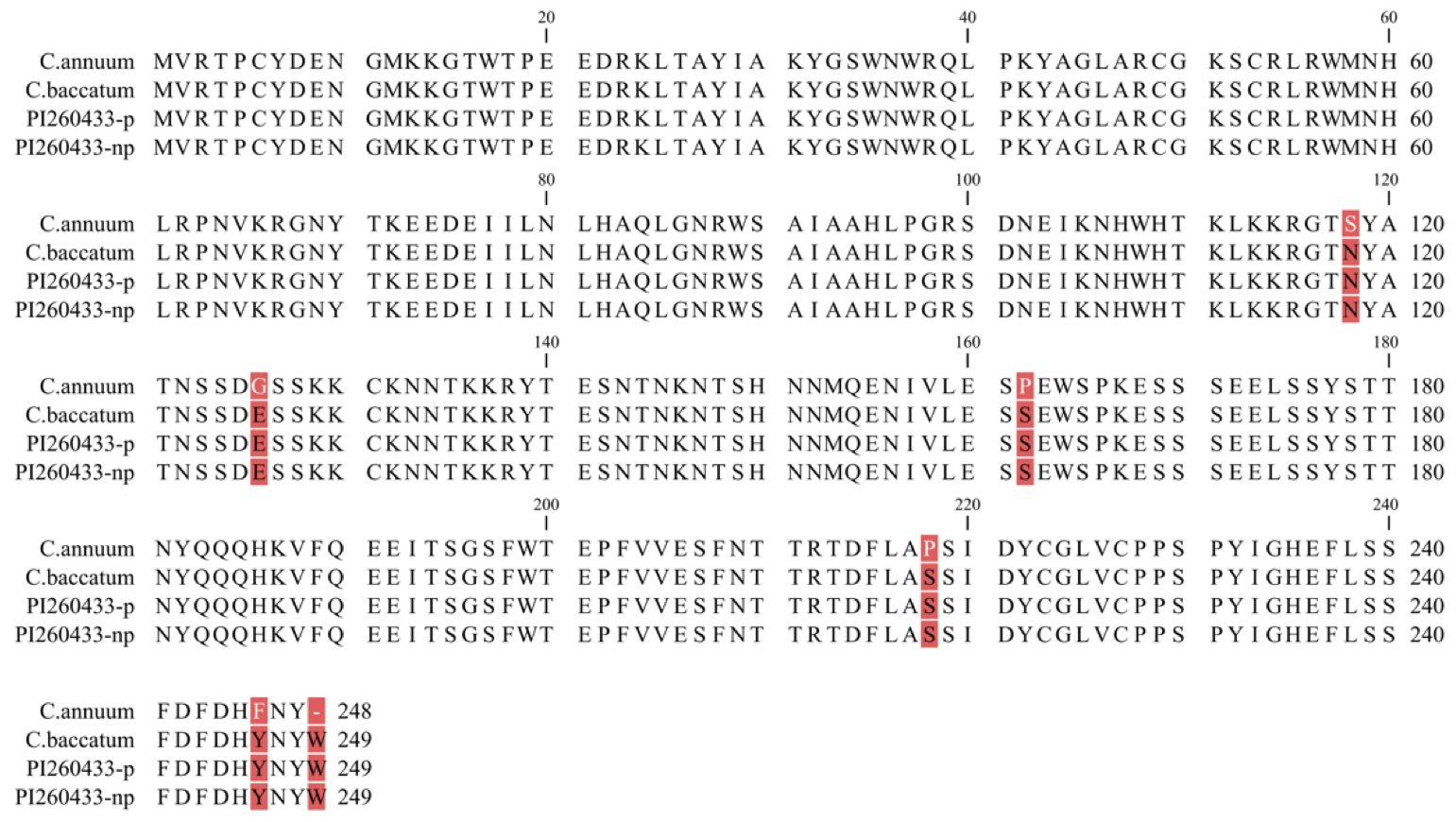

Supplement: Supplementary Figure 5 — Amino acid sequence of the translated protein of Pun3. Amino acid sequences were obtained from the reference genomes of C. annuum ‘Takanotsume’ and C. baccatum ‘PBC81.’ The different residues are marked in white text in black boxes. [file Image_5.tiff]

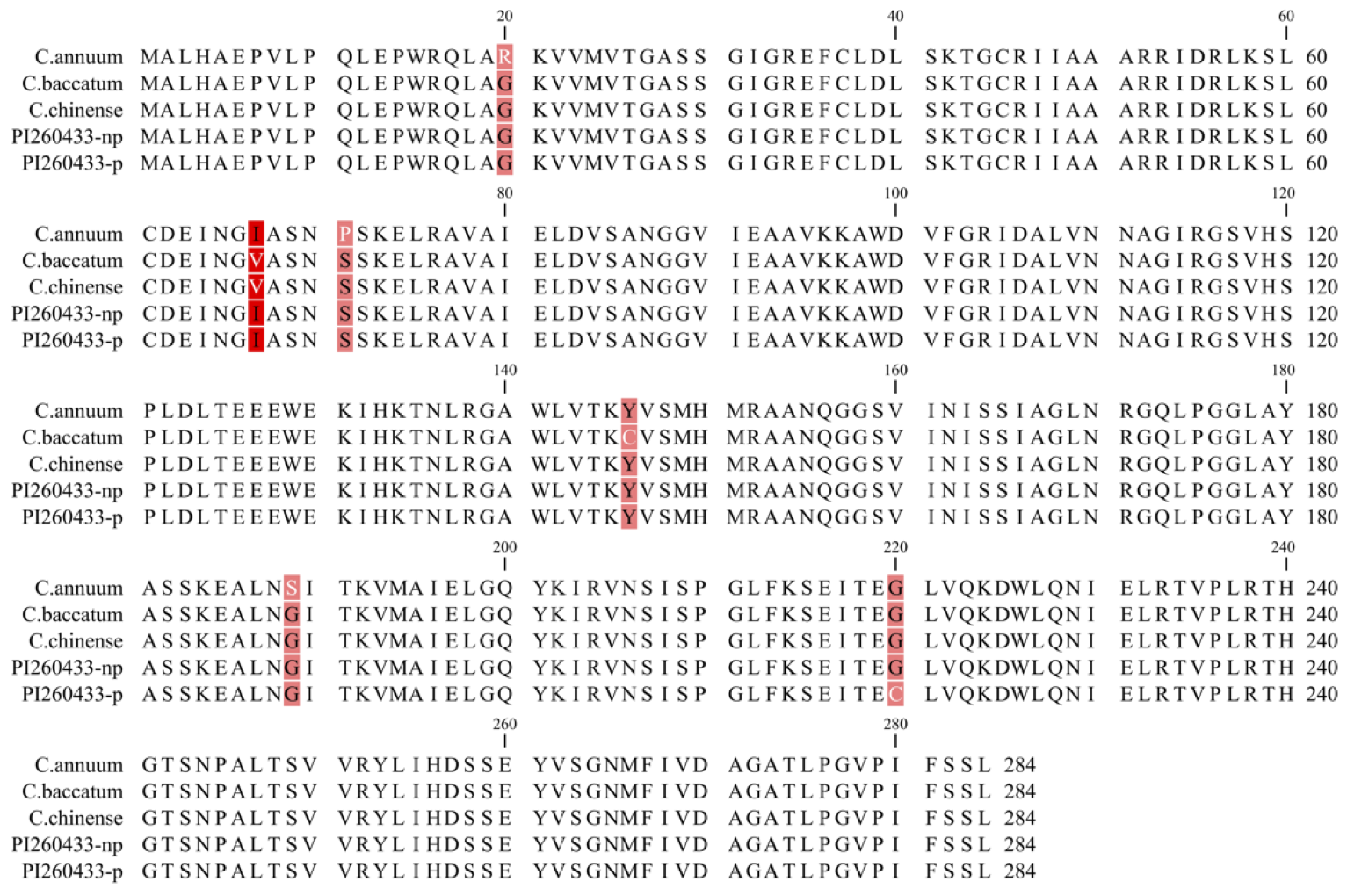

Supplement: Supplementary Figure 6 — Amino acid sequences of the translated protein of CaKR1. Amino acid sequences were obtained from the reference genomes of C. annuum ‘Takanotsume,’ C. baccatum ‘PBC81,’ and C. chinense ‘Habanero.’ The different residues are marked in white text in black boxes. [file Image_6.tiff]
